# CRISPR/Cas9-Mediated Chicken TBK1 Gene Knockout and Its Essential Role in STING-Mediated IFN-β Induction in Chicken Cells

**DOI:** 10.3389/fimmu.2018.03010

**Published:** 2019-01-04

**Authors:** Yuqiang Cheng, Minxiang Lun, Yunxia Liu, Hengan Wang, Yaxian Yan, Jianhe Sun

**Affiliations:** School of Agriculture and Biology, Shanghai Jiao Tong University, Shanghai Key Laboratory of Veterinary Biotechnology, Key Laboratory of Urban Agriculture (South), Ministry of Agriculture, Shanghai, China

**Keywords:** chicken, CRISPR-Cas9, innate immunity, TBK1, IFN-β

## Abstract

TANK-binding kinase 1 (TBK1) is involved in innate immunity, prompting transcriptional induction of type I interferons in response to pathogenic infection. Many studies have focused on mammals but the function of TBK1 in chickens remains poorly defined. CRISPR/Cas9 system has made gene-knockout easy to accomplish. Although CRISPR/Cas9 has been used in chicken cells, low mutation efficiency limits its wide application in chickens. In this study, an effective gene-knockout system was developed based on the CRISPR/Cas9 system in chicken embryonic fibroblast DF-1. Two CRISPR/Cas9 plasmids were constructed, TBK1-g1 and TBK1-g2, which express gRNAs targeting different sequences of the chicken TBK1 gene. After transfection and enrichment with puromycin screening, the mutation rates as assessed via T7E1 assay were 88.05 and 89.55%, respectively, and subsequent sequence analysis showed mutation efficiencies of 86.67 and 93.33%. With the limiting-dilution method, a chTBK1 gene-deficiency monoclonal cell line was obtained and was named DF-1-TBK1-C3. The DF-1-TBK1-C3 cells exhibited normal morphology and maintained stable proliferation ability compared to wild-type cells. The gene-overexpression system and luciferase reporter assay showed that IFN-β induction induced by chSTING was almost completely blocked in DF-1-TBK1-C3 cells. With quantitative real-time PCR, we further confirmed the essential role of chTBK1 in the chSTING-mediated IFN-β induction. At last, the study demonstrated that the chTBK1 knockout system is also applicable in primary chick embryo fibroblasts (CEFs). In this study, an effective gene-knockout system was applied in chickens, a TBK1 gene-deleted DF-1 cell line was successfully created using this system, and with the chTBK1 knockout cells, chTBK1 was revealed to be indispensable in STING-mediated IFN-β activation in chicken cells.

## Introduction

Rapid progress in the understanding of innate immunity has revealed that the innate immune system contains pattern-recognition receptors (PRRs) that can detect specific pathogen-associated molecular patterns (PAMPs). These PAMPS trigger signaling pathways that lead to the transcription of interferons (IFNs), which are crucial in host defense against infection (1). Identified PRRs include Toll-like receptors (TLRs), RIG-I-like receptors (RLRs), and Nod-like receptors (NLRs) (1). The signaling network that triggers IFN production also recruits adaptor proteins, such as TRIF (TIR-domain-containing adapter-inducing IFN-β) and MAVS (mitochondrial antiviral signaling protein). These proteins activate TBK1 to phosphorylate interferon regulatory factor 3 (IRF3), which is indispensable for driving the production of IFN-β (2, 3). Members of the IκB kinase (IKK) family are critical to the inflammatory response. While canonical IKKs activate transcriptional factor NF-κB, TBK1 phosphorylates transcriptional factor IRFs, resulting in their dimerization, cytoplasm-to-nucleus translocation, and DNA binding to stimulate the transcription of IFNs (4, 5).

In our previous study, chSTING was identified as a novel and important IFN mediator that participates in both DNA and RNA recognition in chicken cells (6). In a subsequent study, we found that chTBK1 could interact with chSTING and speculated that chTBK1 might be involved in the regulation of chSTING-triggered IFN-β signaling in chicken cells (7). To verify that chTBK1 plays a role in chSTING-mediated IFN regulation, a chTBK1-knockout cell line is necessary.

Gene editing is important in the study of gene function. Among the gene-editing technologies, CRISPR/Cas9 gene knockout is used to mediate stable gene-knockout. The targeting efficiency of CRISPR/Cas9 (up to 80%) is higher than that of both ZFN and TALEN (8). To date, CRISPR/Cas9 has been applied in human cells and in many other organisms (9, 10). However, its application in chickens is only in the preliminary stages. In the present study, a chTBK1-knockout DF-1 cell line called DF-1-TBK1-C3 was generated using CRISPR/Cas9. The results indicated that this system is a robust tool for chicken genome editing. Using this cell line, we investigated the effects of chTBK1 deletion on chSTING-induced IFN-β production.

## Materials and Methods

### Cell Culture

DF-1, a chicken embryonic fibroblast cell line purchasing from ATCC, was cultured as in our previous study (11). Briefly, DF-1 cells were maintained in high-glucose DMEM (Life Technologies, Grand Island, NY, USA) supplemented with 10% FBS (Life Technologies), and incubated at 37°C in 5% CO_2_. Primary chicken embryo fbroblast cells (CEFs) were prepared from 9-day-old, specifc-pathogen-free (SPF) embryos which were obtained from Merial Vital (Beijing, China) and cultured in high-glucose DMEM supplemented with 10% FBS (Life Technologies), and incubated at 37°C in 5% CO_2_.

### Targeting Strategy

According to the chTBK1 sequence obtained from the NCBI database, we designed gRNA sequences respectively targeting CDS1 and CDS2 of chTBK1, known as gRNA1: GTCTGACATTCTAGGACA and gRNA2: TGAACATCCACGGGGCGA. By synthesizing the oligo-DNAs of these gRNAs and annealing them to a T7 promoter-driven Cas9 vector and a U6 promoter-driven gRNA vector, two gRNA-expressing plasmids, TBK1-g1 and TBK1-g2, were formed. The construction of the established plasmids was then confirmed by sequence analysis.

### Cell Transfection and Drug Selection

DF-1 cells were seeded in 6-well plates for further transfection using Lipofectamine 2000 (Invitrogen, Carlsbad, CA, USA). The dose used for each well was 3 μg of the CRISPR/Cas9 vectors and 6 μL of the transfection reagent, and the transfection procedure was performed according to the manufacturer's instructions. After a 48-h recovery period, the cells were supplemented with 2 μg/mL of puromycin (Sigma) in the culture medium for 12 days until successful selection. During this period, green fluorescence was regularly observed due to the existence of GFP in the plasmid construction, to detect the percentage of transfected cells. Cells were then collected and genomic DNA was extracted for knockout-efficiency analysis with the T7 endonuclease I assay.

### T7 Endonuclease I Assay (T7E1)

T7 endonuclease I (T7E1) recognizes and cleaves non-perfectly matched DNA, which makes it useful in knockout-efficiency analysis and knockout confirmation. The primers listed in Table 1 were used first for polymerase chain reaction (PCR) amplification to obtain the DNA fragments, including the target sites. T7E1 buffer was then mixed in and annealed at 95°C for 5 min, followed by gradual temperature reduction to 25°C at a rate of 0.1°C/s. The heteroduplex amplicons were subsequently treated with T7E1 endonuclease (New England Biolabs) for 30 min at 37°C and then analyzed with 1.5% agarose gel electrophoresis. To further confirm knockout efficiency, the PCR fragments were cloned and sequenced.

**Table 1:** Primers used in sequence analysis for detection of indels.

**Target locus**	**Sequences**	**PCR product (bp)**
TBK1-g1	F: TGTTGAGTTTGGTTTCGGTTTTGTGT	500
	R: GTAACTGCATAGAACTCTAATTCAGTG	500
TBK1-g2	F: GTTAAATCTGGGTTTCCATTTTTCC	630
	R: CTTCTTGAGATGGCTTTGAAGAAAC	630

### Culturing of Single DF-1 Cells and Genomic DNA Sequencing

The cells were serially diluted onto 96-well plates to obtain individual cells for further cell-line establishment. When the cells in each well reached confluency, they were collected and subjected to direct PCR with the Phanta Master Mix (Vazyme, Jiangsu, China) using specific primer sets (Table 1). The PCR product encompassing the CRISPR/Cas9 target sites was cloned into a pEASY-Blunt Cloning vector (TransGen Biotech, Beijing, China) and the positive colonies were sent to Beijing Genomics Institute (Shanghai, China) for sequencing.

### Western Blot

Whole-cell extracts were prepared as described previously (11). The cell lysates were eluted with 6 × SDS loading buffer (TransGen) and boiled for 10 min. Proteins were separated by sodium dodecyl sulfate-polyacrylamide gel electrophoresis (SDS-PAGE) and transferred onto 0.45-μm polyvinylidene fluoride membranes (PVDF, Millipore®, MA, USA). The membranes were incubated with primary mouse anti-TBK1 antibodies that can detect TBK1 in chickens. The membranes were then incubated with HRP-conjugated goat anti-rabbit antibody (Jackson, West Grove, PA, USA). Images were collected with a Tanon 5200 imaging system (Tanon, Shanghai, China). β-Tubulin expression levels were measured in parallel to serve as controls.

### Quantitative Real-Time PCR

The RNA was extracted using HP Total RNA kits (OMEGA, Guangzhou, China) and reverse transcribed into cDNA using random hexamer primers and Moloney murine leukemia virus reverse transcriptase (Promega, Madison, WI, USA). The obtained cDNA was amplified in 20 μL reactions using the ABI 7500 real-time PCR system using oligonucleotide primers in Table 2. Relative expression levels for tested mRNAs were determined using β-actin as an internal reference using comparative Ct (2^−ΔΔCt^) method.

**Table 2:** Primers used for Quantitative real-time PCR.

**Target gene**	**Primer name**	**Sequence of oligonucleotide (5′-3′)**
IFN-β	qIFN-β-F	CCTCAACCAGATCCAGCATT
	qIFN-β-R	GGATGAGGCTGTGAGAGGAG
Mx-1	qMx-1-F	GTTTCGGACATGGGGAGTAA
	qMx-1-R	GCATACGATTTCTTCAACTTTGG
PKR	qPKR -F	TGCTTGACTGGAAAGGCTACT
	qPKR -R	TCAGTCAAGAATAAACCATGTGTG

### CCK8 Assay

To assess the proliferation of the DF-1 and DF-1-TBK1-C3 cells, a Cell Counting Kit-8 (CCK8) assay was conducted. The cells were seeded individually on a 96-well plate (4000 cells/well) and 10 μl of the CCK8 reagent was added. Signals were measured at an absorption wavelength of 450 nm at 3, 24, 48, 72, 84, 96, 108, and 120 h after seeding.

### Dual-Luciferase Reporter Assay

Cells were plated onto 24-well plates and transiently transfected with reporter plasmid pGL-chIFN-β-Luc and control Renilla luciferase, along with the indicated stimulus plasmids. Twenty-four hours post-transfection, the cells were lysed and luciferase activity was measured using a dual-reporter luciferase assay kit (Promega) according to the manufacturer's instructions. All reporter assays were repeated at least three times.

## Results

### Complete Construction of CRISPR/Cas9 Vector

**Figure 1:**
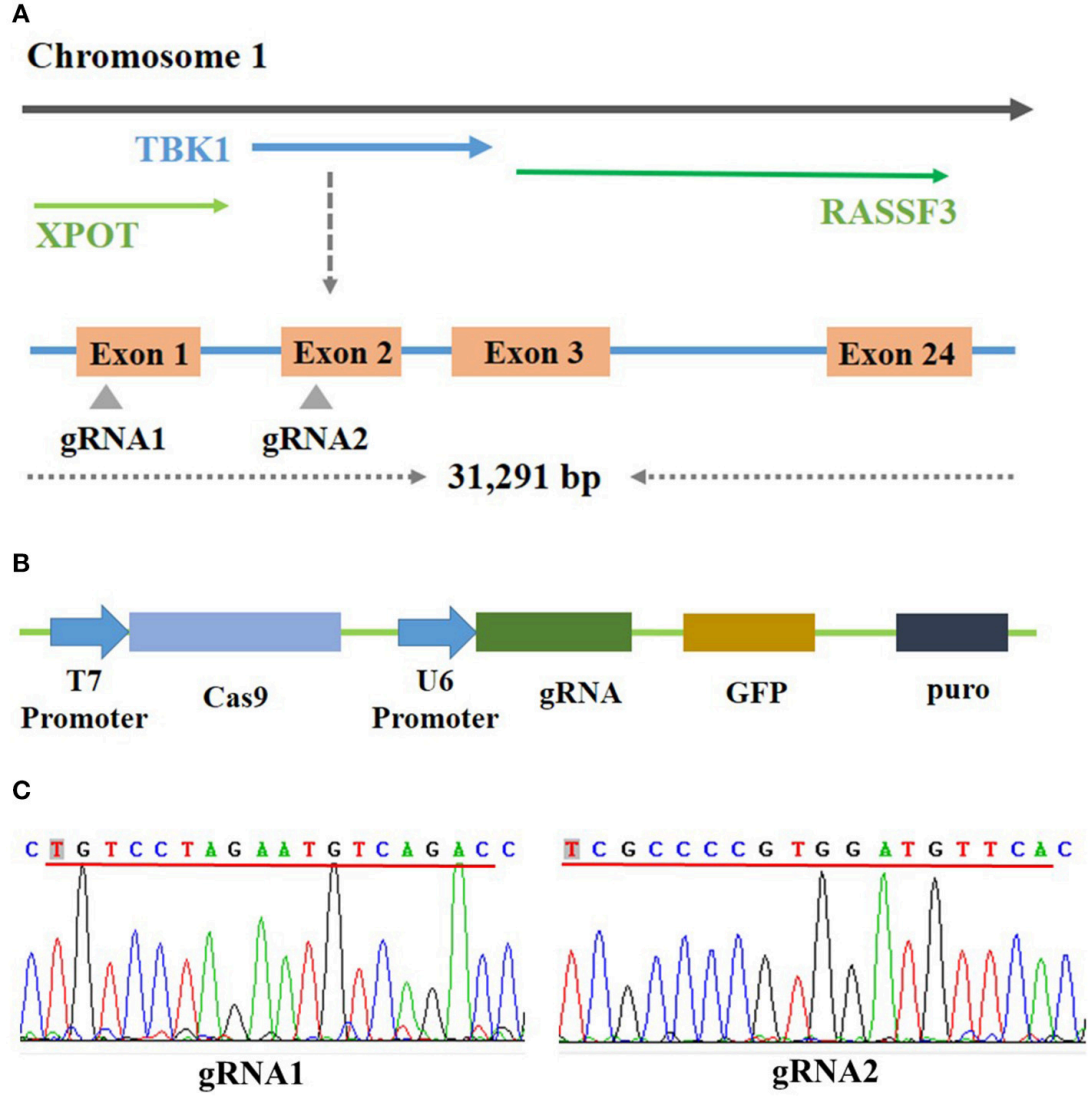
Construction of CRISPR/Cas9 vector. **(A)** Schematic diagram of chicken TBK1 gene and target-site position. Two target sites were designed based on the sequence at exons 1 and 2. **(B)** Schematic diagram of CRISPR/Cas9 vector used in this study. **(C)** Sequencing results showing that gRNA1 and gRNA2 have been completely connected to the CRISPR/Cas9 vector.

**Figure 2:**
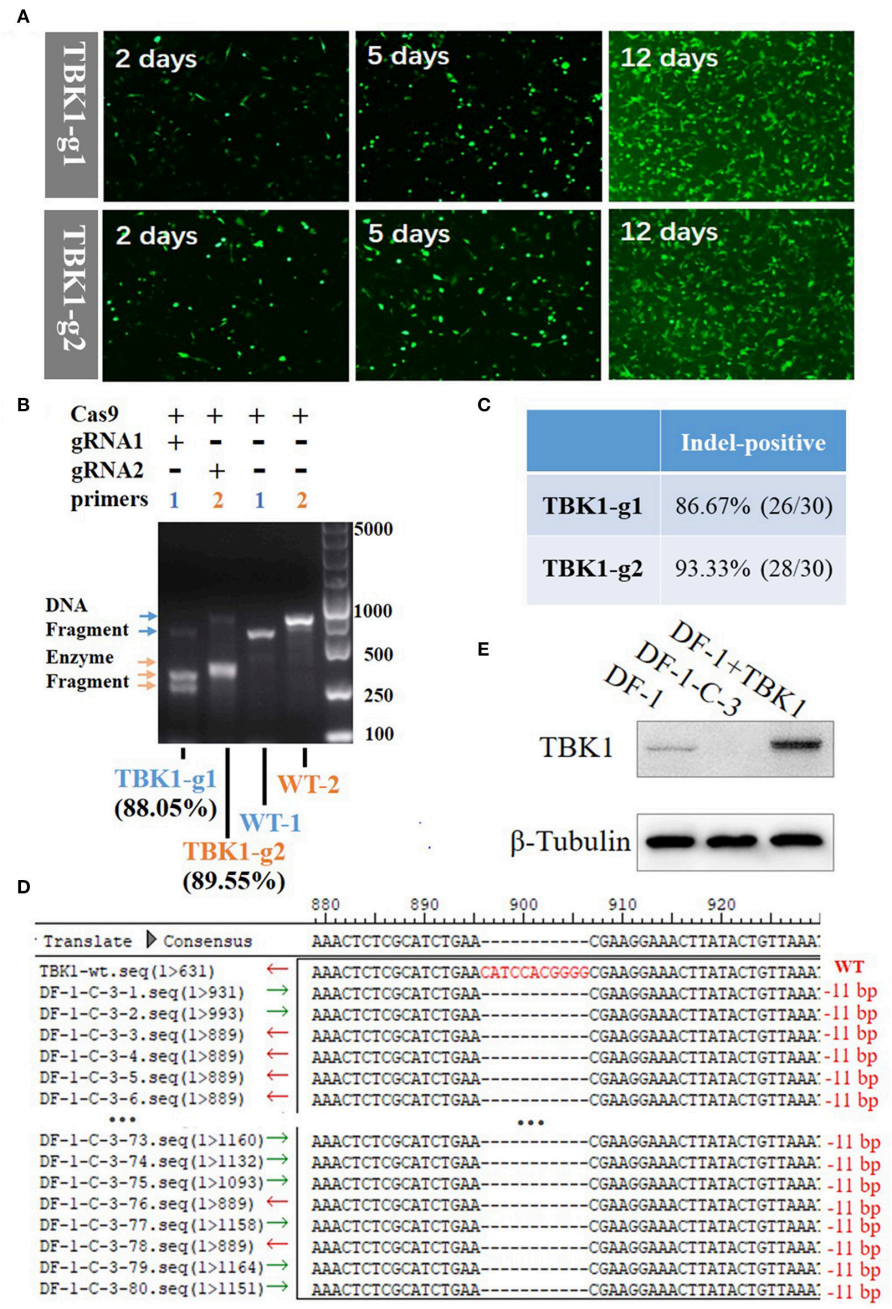
Generation and identification of chTBK1 knockout DF-1 cell line, DF-1-TBK1-C3. **(A)** Mutant cell enrichment using puromycin screening. **(B)** Results of T7EI assay showing high knockout efficiency of both gRNA-1 and gRNA-2. **(C)** Evaluation of knockout efficiency of TBK1-g1 and TBK1-g2 by cloning and sequencing. **(D)** Sequencing results of targeted regions of DF-1-TBK1-C3 monoclonal cells. Alignment of DNA sequences between wild-type and DF-1-TBK1-C3 cells showed homozygous mutation with an 11-bp deletion. **(E)** The chTBK1 protein was not detectable in DF-1-TBK1-C3 cells by Western blot analysis.

Bioinformatics analysis showed that the chicken TBK1 gene is located on chromosome 1 and contains 31,291 bp and 24 exons (Figure 1A). The sequencing results showed the successful construction of the two CRISPR/Cas9 plasmids that express gRNA and target two unique sequences present on exons 1 and 2 of chicken TBK1 (Figures 1B,C). These vectors efficiently expressed GFP in DF-1 cells (Figure 2A).

### CRISPR/Cas9-Mediated Gene Knockout in DF-1 Cells

By transfecting cells with the CRISPR-Cas9 vectors, drug-selectable DF-1 cells were established. GFP-positive cells were screened by supplementing the culture medium with 2 μg/mL puromycin for 12 days. After puromycin selection, the un-transfected cells were dead, leaving only the GFP-positive cells (Figure 2A). The T7E1 assay showed that the DF-1 fibroblasts after puromycin selection had insertion or deletion (indel) mutations at the target locus, with 88.05 and 89.55% efficiency in the TBK1-g1 and TBK1-g2 transfected cells, respectively (Figure 2B). Clone sequencing showed a knockout efficiency of 86.67% (26/30) and 93.33% (28/30) in the TBK1-g1 and TBK1-g2 transfected cells (Figure 2C and Supplementary Figures 1A,B), respectively, similar findings to those of the T7E1 assay (Figure 2B). The results showed that both gRNAs could induce indels in the TBK1 target region, while gRNA2 showed a slightly higher cleavage efficiency than gRNA1 (Figures 2B,C). Therefore, gRNA2 was chosen for subsequent TBK1 gene-targeting.

### chTBK1 Homozygous Mutation Clone

The limiting-dilution method was used to obtain a chTBK1 gene-deficiency monoclonal cell line with the TBK1-g2 transfected cells. Ten monoclonal cell lines were obtained and DNA was extracted. PCR and clone sequencing revealed that the DF-1-TBK1-C3 clone is a homozygous mutation with an 11-bp nucleotide deletion, resulting in a frame-shift mutation (Figure 2D). Western blot showed that chTBK1 protein expression was not detected in the homozygous mutation clone DF-1-TBK1-C3 (Figure 2E).

### Knockout of chTBK1 to Block chSTING-Mediated IFN-β Production

**Figure 3:**
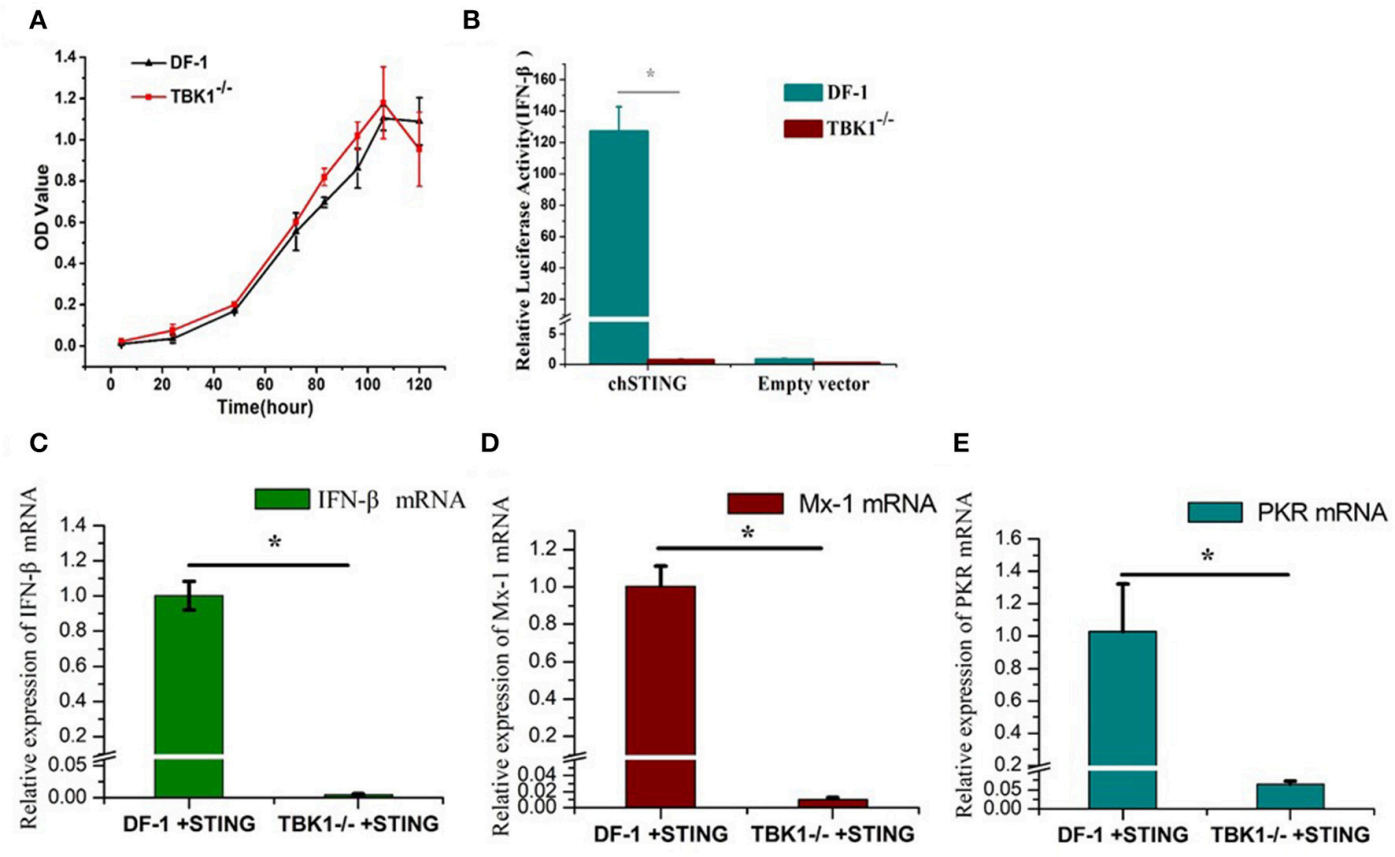
Knockout of chTBK1 to Block chSTING-mediated IFN-β Production. **(A)** Proliferation of DF-1-TBK1-C3 cell line (TBK1^−/−^) using CCK8 assay. **(B)** Knockout of chTBK1 blocks IFN-β production induced by chSTING overexpression. DF-1 or TBK1-C-3 cells were cotransfected with 100 ng/well chSTING or empty vector with 100 ng/well pGL-chIFN-β-Luc with 20 ng/well pRL-TK. Luciferase assays were performed 24 h after cotransfection. **(C–E)** DF-1 or TBK1-C-3 cells were transfected with 100 ng/well chSTING plasmid, 24 h after transfection, the cells were harvest, and IFN-β **(C)**, Mx-1 **(D)**, and PKR **(E)** mRNAs were analyzed by qRT-PCR. Asterisks indicate a significant difference (*p* < 0.05) as determined by Student's *t*-test.

The CCK8 assay was used to detect the proliferation of wild-type and chTBK1 gene deficient cells DF-1-TBK1-C3. No significant difference was detected at any time-point (Figure 3A), indicating that chTBK1 knockout did not influence the proliferation of DF-1 cells and therefore chTBK1 knockout cells can be used for subsequent studies. Using the DF-1-TBK1-C3 cells, the function of chTBK1 in innate immunity was assessed. With a chicken IFN-β luciferase assay, we found that the IFN-β promoter activity induced by chSTING overexpression was almost abolished in chTBK1-deficient DF-1 cells (Figure 3B). We then detected the mRNA expression of the IFN-β and the IFN-related genes by chSTING in both DF-1 and DF-1-TBK1-C-3. The results showed that the mRNA of IFN-β induced by chSTING was much lower in the chTBK knockout cells than in the normal DF-1 cells (about 205 times lower; Figure 3C). The IFN-stimulated genes Mx-1 and PKR, whose expression was regulated by IFNs, are indicators of IFNs. In the present study, both Mx-1 and PKR mRNAs induced by chSTING were significantly inhibited in chTBK1 knockout cells (Figures 3C,D), and this further confirmed that chTBK1 plays an essential role in chSTING-mediated IFN-β induction.

### The chTBK1 Knockout Cell Line Was Stable

**Figure 4:**
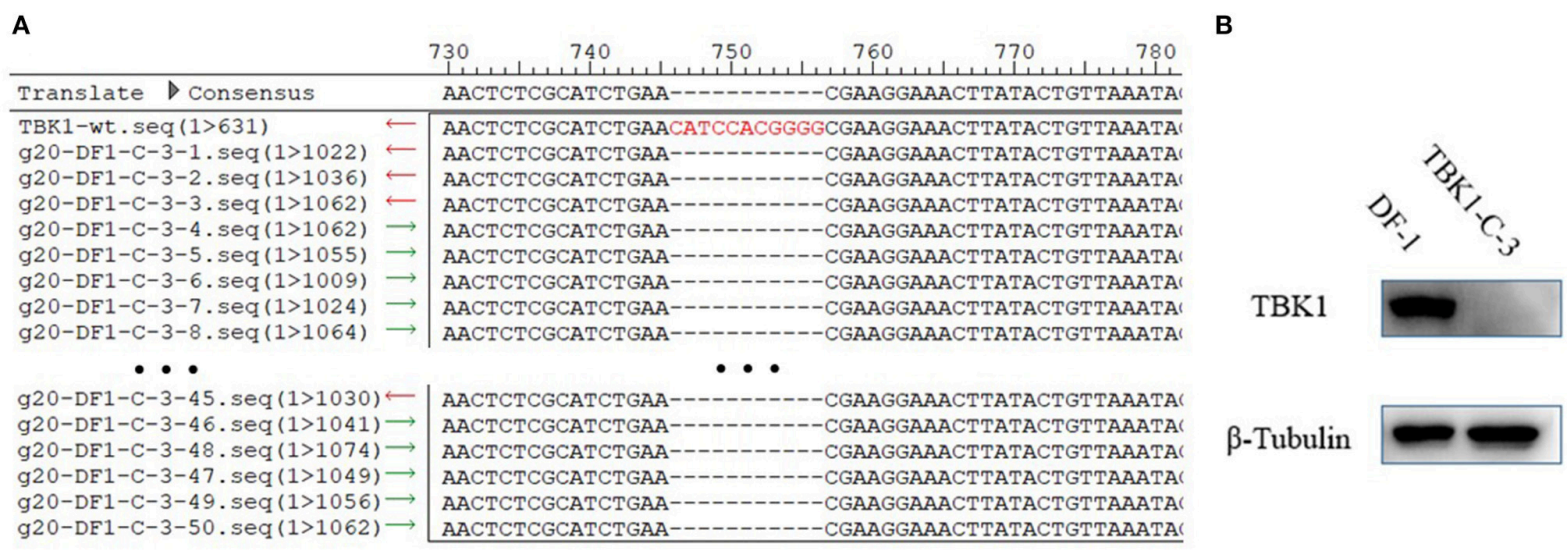
The chTBK1 knockout cell line was stable. **(A)** Sequencing results for the regions of g20-DF-1-TBK1-C3 cells targeted using CRISPR/Cas9. Alignment of DNA sequences between the wild-type and the g20-DF-1-TBK1-C3 cells showed homozygous mutation with an 11-bp deletion. **(B)** DF-1 cells and g20-DF-1-TBK1-C3 cells were lysed and analyzed using Western blot.

To test the stability of DF-1-TBK1-C3 cells, the DF-1-TBK1-C3 cells were passaged for 20 generations (g20-DF-1-TBK1-C3), and the CRISPR/Cas9 target site and the protein expression of chTBK1 were detected using clone sequencing and Western blot, respectively. As Figure 4A shows, the 11-bp nucleotide deletion still existed on the genome of g20-DF-1-TBK1-C3. The Western blot showed that the chTBK1 band was undetectable in g20-DF-1-TBK1-C3 cells (Figure 4B). These results indicated that the chTBK1 knockout cell line is stable, or at least that it remains stable for at least 20 generations.

### The chTBK1 Knockout System Was Applicable in CEFs

**Figure 5:**
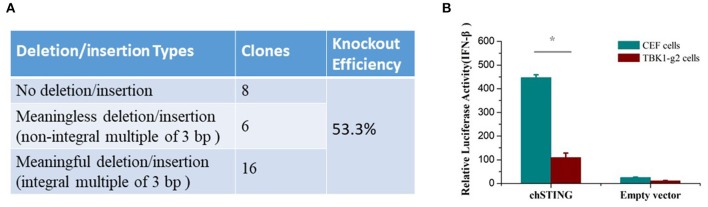
The chTBK1 knockout system was applicable in CEFs. **(A)** Knockout efficiency of CEF cells transfected with TBK1-g2 were evaluated by cloning and sequencing. The knockout efficiency was calculated as “meaningful deletion or insertion clones/all clones.” **(B)** CEF or CEF-TBK1-g2 cells were cotransfected with either 100 ng/well of chSTING or empty vector with luciferase reporter plasmids. Luciferase assays were performed 24 h after cotransfection. Asterisks indicate a significant difference (*p* < 0.05) as determined by Student's *t*-test.

To determine whether the chTBK1 gene knockout system can be used in chicken primary cells, the CRISPR-Cas9 vector containing TBK1-g2, which showed a higher knockout efficiency in DF-1 cells, was transfected into CEFs. After puromycin selection, the chTBK1 knockout efficiency was found to be more than 50% in the CEFs transfected with TBK1-g2 by sequencing the genome DNA containing the target site (Figure 5A). This indicated that the chTBK1 knockout system mediated with CRISPR/Cas9 was also applicable in CEFs.

Given that the primary CEFs can be cultured only for 3–5 generations under our current culturing conditions, the chTBK1 gene-deficiency monoclonal cell line cannot be obtained using a limiting dilution method, which requires several passages. Therefore, we used the 53.3% chTBK1 knockout CEFs, which were named CEF-TBK1-g2, for the subsequent function study of chTBK1 in CEFs. The results showed that the IFN-β promoter activity induced by chSTING overexpression was also obviously suppressed in the CEF-TBK1-g2 cells (Figure 5B). This further confirmed the function of chTBK1 in IFN-β production mediated by chSTING in chickens.

## Discussion

As a cutting-edge gene-editing technique, CRISPR/Cas9 has been used on human cells and those of many other organisms. However, application of CRISPR/Cas9 in chickens is only in the preliminary stages. One barrier to its application in chicken cells is low mutation efficiency. Previous studies showed that mutation efficiency is usually low, even less than 30%, in chicken cells (12, 13).

In the present study, CRISPR/Cas9 plasmids were constructed to target exons 1 and 2 of chicken TBK1. After transfection and enrichment using puromycin screening, the mutation rates of chTBK1 were greater than 88.05% on the T7E1 assay and greater than 86.67% on the sequencing analysis. To our knowledge, this is the highest mutation efficiency introduced by CRISPR/Cas9 in chicken cells (12, 14). A high mutation efficiency is helpful for screening of gene-deficiency monoclonal cell lines. With the limiting-dilution method, a homozygous mutation monoclonal DF-1 cell line was successfully obtained. After that we further test whether the chTBK1 knockout system can be used in chicken primary CEFs. Because of CEFs can be cultured only for limited generations under our current culturing conditions, we only obtained 53.3% chTBK1 knockout CEFs with the chTBK1 knockout system. It is at least demonstrated that the chTBK1 knockout system was applicable in CEFs. It should be noted that the chTBK1-g2 knockout efficiency in CEFs (53.3%) seems to be much lower than that of DF1 cells (93.3%). We speculate that it may be caused by the low transfection efficiency of CEFs and the insufficient puromycin selection. Therefore, our results demonstrated that the CRISPR/Cas9 system can be used as a robust tool for chicken genome editing.

Microscopic observation and CCK8 assays showed that the DF-1-TBK1-C3 cell line exhibits normal morphology and maintains stable proliferation ability compared to wild-type cells, indicating that this cell line can be used for further studies. Functional studies showed that chSTING-induced IFN-β production was almost abolished in the DF-1-TBK1-C3 cells (Figures 3B–E). With the 53.3% chTBK1 knockout CEFs obtained in this study, we further confirmed the crucial role of chTBK1 in the chSTING-mediated IFN-β induction (Figure 5). These indicates that chSTING-mediated IFN-β production is highly dependent on chTBK1, and that chTBK1 is indispensable for this process in chicken cells.

In conclusion, a chTBK1-knockout DF-1 cell line (DF-1-TBK1-C3) was generated using the CRISPR/Cas9 system to efficiently target chicken cells. Analysis of the DF-1-TBK1-C3 cells revealed that chTBK1 is indispensable for chSTING-mediated IFN-β regulation. This study provides an example for the application of CRISPR/Cas9 in chicken cells and a model for the molecular regulation mechanism of the chTBK1 gene *in vitro*. The findings also revealed the function of chTBK1 in chSTING-induced IFN-β production.

## Author Contributions

JS and YC designed the experiment; ML, YC, and YL performed the experiments; YY and HW analyzed the experimental results and developed analysis tools; YC and ML wrote the paper.

### Conflict of Interest Statement

The authors declare that the research was conducted in the absence of any commercial or financial relationships that could be construed as a potential conflict of interest.

## Supplementary Material

Supplementary Figure 1 Sequencing results of targeted regions of DF-1 cells transfected with CRISPR/Cas9 vectors TBK1-g1 **(A)** and TBK1-g2 **(B)** after screening with puromycin.
