# Supplementary figures and images for: CRISPR/Cas9-Mediated Chicken TBK1 Gene Knockout and Its Essential Role in STING-Mediated IFN-β Induction in Chicken Cells
Source: Front Immunol. 2019 Jan 4;9:3010. doi: 10.3389/fimmu.2018.03010 (PMC6328437; doi:10.3389/fimmu.2018.03010)

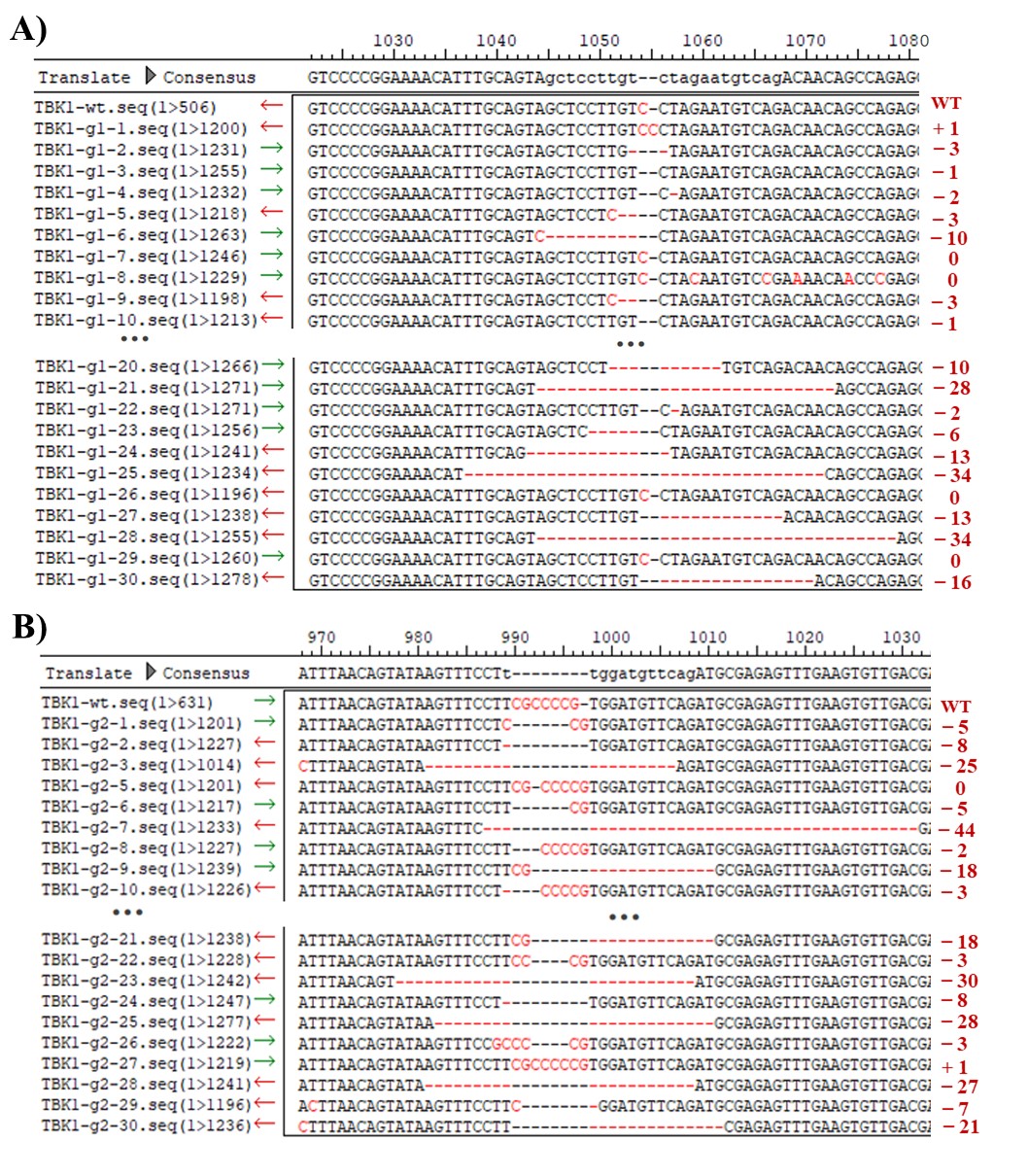

Supplement: Supplementary Figure 1 — Sequencing results of targeted regions of DF-1 cells transfected with CRISPR/Cas9 vectors TBK1-g1 (A) and TBK1-g2 (B) after screening with puromycin. [file Image_1.JPEG]
